# ACAT1‐Mediated ME2 Acetylation Drives Chemoresistance in Ovarian Cancer by Linking Glutaminolysis to Lactate Production

**DOI:** 10.1002/advs.202416467

**Published:** 2025-02-14

**Authors:** Cuimiao Zheng, Hao Tan, Gang Niu, Xi Huang, Jingyi Lu, Siqi Chen, Haoyuan Li, Jiayu Zhu, Zhou Zhou, Manman Xu, Chaoyun Pan, Junxiu Liu, Jie Li

**Affiliations:** ^1^ Department of Obstetrics and Gynecology The First Affiliated Hospital Sun Yat‐sen University Guangzhou 510080 China; ^2^ Department of Biochemistry and Molecular Biology Zhongshan School of Medicine Sun Yat‐sen University Guangzhou 510080 China; ^3^ Advanced Medical Technology Center The First Affiliated Hospital Zhongshan School of Medicine Sun Yat‐sen University Guangzhou 510080 China

**Keywords:** ME2, acetylation, lactylation, platinum resistance

## Abstract

Lactate derived from aerobic glycolysis is crucial for DNA damage repair and chemoresistance. Nevertheless, it is frequently noted that cancer cells depend on glutaminolysis to replenish essential metabolites. Whether and how glutaminolysis might enhance lactate production and facilitate DNA repair in cancer cells remains unknown. Here, it is shown that malate enzyme 2 (ME2), which metabolizes glutamine‐derived malate to pyruvate, contributes to lactate production and chemotherapy resistance in ovarian cancer. Mechanistically, chemotherapy reduces the expression of glucose transporters and impairs glucose uptake in cancer cells. The resultant decrease in intracellular glucose levels triggers the acetylation of ME2 at lysine 156 by ACAT1, which in turn potentiates ME2 enzyme activity and facilitates lactate production from glutamine. ME2‐derived lactate contributes to the development of acquired chemoresistance in cancer cells subjected to prolonged chemotherapy, primarily by facilitating the lactylation of proteins involved in homologous recombination repair. Targeting ACAT1 to inhibit ME2 acetylation effectively reduced chemoresistance in both in vitro and in vivo models. These findings underscore the significance of acetylated ME2‐mediated lactate production from glutamine in chemoresistance, particularly under conditions of reduced intracellular glucose within cancer cell, thereby complementing the Warburg effect and offering new perspectives on the metabolic links to chemotherapy resistance.

## Graphical abstract

Chemotherapy reduces glucose transporter expression and glucose uptake in cancer cells, leading to decreased pyruvate levels. This decline prompts ACAT1‐mediated acetylation of ME2 at Lys156, enhancing ME2 activity and promoting lactate production from glutamine. This ME2‐dependent pathway is crucial for DNA repair protein lactylation and chemoresistance under conditions of low intracellular glucose.

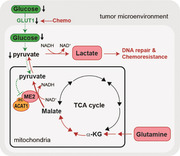

## 1 Introduction

Tumor tissue is characterized by a substantial increase in lactate production, similar to fermentation, even when the oxygen supply is sufficient, a phenomenon termed the “Warburg effect”.^[^ ^1^ ^]^ While the generation of lactate from pyruvate is coupled with the rapid recycling of NAD^+^ from NADH to facilitate catabolic reactions in cancer cells, lactate itself has been shown to play important roles in diverse biological processes of cancer development.^[^ ^2^ ^]^ In the tumor microenvironment, excessive lactate shunts energy sources between different cell populations, causes extracellular acidification, and modulates the immune response.^[^ ^3^ ^]^ Lactate can regulate the cell cycle of rapidly growing cells by directly binding and remodeling the anaphase‐promoting complex.^[^ ^4^ ^]^ Lactate also serves as a lactyl donor for protein lactylation. Recent works have shown that lactate promotes DNA repair and resistance to DNA‐damaging chemotherapy by lactylating DNA repair proteins, including MRE11,^[^ ^2^ ^]^ NBS1,^[^ ^5^ ^]^ and XRCC1.^[^ ^6^ ^]^ However, although lactate accumulation in tumors is important for tumor development, the metabolic pathway controlling lactate production remains unclear.

Aerobic glycolysis, or the Warburg effect, represents an important avenue for lactate production from glucose in cancer cells. However, owing to various factors such as the inadequate and dysregulated vasculature, the glucose level in solid tumors is significantly lower than that in the original normal tissue; thus, the glucose availability for cancer cells is extremely limited.^[^ ^7^ ^]^ Furthermore, studies on nutrient partitioning have revealed that myeloid cells have the greatest capacity to take up intratumoral glucose, followed by T cells, whereas cancer cells have the highest uptake of glutamine in a variety of cancer models.^[^ ^8^ ^]^ Indeed, glutaminolysis has been shown to play an important role in tumor growth and metastasis and therapeutic resistance.^[^ ^9^ ^]^ However, whether and how cancer cells use glutaminolysis to promote lactate production and tumor progression are largely unexplored.

In the present study, using an in vivo acquired chemoresistance mouse model,^[^ ^10^ ^]^ we found that malic enzyme 2 (ME2) is important for lactate production from glutaminolysis and confers chemoresistance. We first approached this question by performing proteome‐wide acetylation analysis of chemoresistant ovarian tumors, considering that acetyl‐CoA is a central metabolite linking the metabolism of both glucose and glutamine with cell signaling.^[^ ^11^ ^]^ We determined that ME2 was highly acetylated at K156 and thus strongly activated in cancer cells, resulting in chemoresistance.

ME2 generates pyruvate from malate, a TCA intermediate, via NAD(P)^+^ in mitochondria.^[^ ^12^ ^]^ Previous studies have revealed the multiple roles of ME2 in promoting tumor progression. ME2 has been shown to regulate the epigenetic state of cells through NAPDH.^[^ ^13^ ^]^ In addition, ME2 promotes the production of 2‐hydroxyglutarate to stabilize the oncogenic mutant p53 protein by altering glutaminolysis and NAPDH production.^[^ ^14^ ^]^ By producing NADPH to maintain cellular redox homeostasis, ME2 has also been shown to play an essential role in MYC‐driven T‐cell lymphomagenesis.^[^ ^15^ ^]^ The involvement of ME2 in glutaminolysis also contributes to the regulation of the cell cycle in cancer and the antitumor immunity of CD8^+^ T cells through the regulation of α‐KG^[^ ^16^ ^]^ and fumarate,^[^ ^17^ ^]^ respectively. While these studies have extensively explored the changes in NAPDH and TCA intermediate levels caused by ME2, the metabolic fate of pyruvate produced by ME2 remains unknown.

The current study revealed that pyruvate derived from ME2 contributes critically to lactate production. Intriguingly, we found that long‐term chemotherapy treatment suppressed the expression of glucose transporter and inhibited glucose uptake in cancer cells. Low level of intracellular glucose promotes ME2 binding to acetyltransferase ACAT1 but dissociates it from SIRT4 to increase the acetylation of ME2 at K156. Acetylated ME2 significantly contributes to lactate production in cancer cells, which is critical for DNA damage repair and cell survival. Targeting ME2 acetylation with a small‐molecule inhibitor of ACAT1 attenuated acquired chemoresistance in vitro and in vivo. Our findings highlight the rewired glutamine metabolism mediated by ME2 in tumor cells as a crucial regulator of lactate production and the therapeutic potential of targeting ME2 acetylation to promote chemotherapy in ovarian cancer.

## 2 Results

### 2.1 Acetylation of ME2 at Lysine 156 is Associated with Platinum‐Based Chemotherapy Resistance

**Figure 1:**
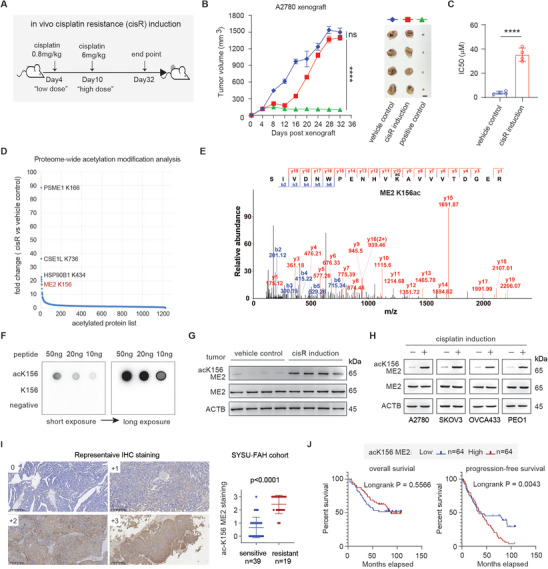
Acetylation of ME2 at the lysine 156 site is associated with platinum resistance. A–C) Development of a cisplatin‐resistant model in vivo. A) Illustration of the cisplatin‐resistant tumor induction schedule. Mice were treated with 2 doses of cisplatin at low (0.8 mg kg^−1^) and high (6 mg kg^−1^) concentrations. B) Tumor volumes in the vehicle control group (PBS treatment), cisplatin‐resistant induction (cisR) group, and positive control group (3 mg kg^−1^ cisplatin treatment, twice per week), 2‐way ANOVA. C) Cisplatin IC50 values of cancer cells harvested from the vehicle control group and the cisplatin‐resistant induction (cisR) group. Data are presented as the means ± SDs and were analyzed by Student's t test. D) Proteome‐wide acetylation analysis of xenograft tumors collected from the vehicle control group and the cisplatin‐resistant induction (cisR) group. ME2 K156 is highlighted in red. E) Mass spectrometry showing the mutation of the Malic enzyme 2 (ME2) K156 site in cancer cells from the cisplatin‐resistant induction (cisR) group. F) Dot blot verifying the specificity and potency of the custom antibody against acK156 of ME2. G) Western blot analysis of ME2 acK156 in the cancer cells harvested from mice in the vehicle control group and the cisplatin‐resistant induction (cisR) group. H) Western blot analysis of ME2 acK156 in the indicated cells. For cisplatin induction, the cells were continuously treated with 1 µg ml^−1^ cisplatin for 7 days. I) Immunohistochemistry analyses of ME2 acK156 in primary tumor samples collected from patients treated with platinum‐based therapy. Tumors from patients who were sensitive (blue, n = 39) or resistant (red, n = 19) to platinum‐based therapy were compared. Student's t test was used. Representative images of ME2 acK156 IHC staining at scores of 0, +1, +2, and +3 are shown. Scale bars, 200 µM. J) Overall survival (right panel) and progression‐free survival (left panel) of ovarian cancer patients who were equally stratified by the level of ME2 acK156 intensity. The ME2 acK156 intensity was determined via immunohistochemistry analyses followed by ImageJ quantification. The data shown are representative of 3 independent biological experiments for (G) and (H). ****P < 0.0001, ns: not significant. See also Figure S1 (Supporting Information).

To better understand the relationship between cancer metabolism and chemotherapy resistance, we established an in vivo mouse model of cisplatin resistance (cisR) induction by administering two sequential “low” and “high” doses of cisplatin to mice bearing A2780 human ovarian cancer cells as we previously described^[^ ^10^ ^]^ (**Figure** **1**A). Tumors were harvested for cancer cell isolation and cisplatin IC50 confirmation when the tumor volume was not significantly different between the vehicle control and cisR induction groups (Figure 1B,C). Considering that acetyl‐CoA is positioned at the crossroads of cell signaling and metabolism, being both the acetyl donor for acetylation reactions and the central metabolic intermediate from the metabolism of glucose, fatty acids, and amino acids, we carried out a proteome‐wide acetylation analysis to evaluate the change in protein acetylation in acquired cisplatin‐resistant tumors compared with treatment‐naïve tumors. The top candidates include proteins previously identified as important for controlling protein stability and proteasome degradation,^[^ ^18^ ^]^ including HSP90B, CSE1L and PSME1. We identified malic enzyme 2 (ME2) as one of the top cisplatin‐induced factors that results in an ≈18‐fold increase in protein acetylation at the lysine 156 (K156) site (Figure 1D; Table S1, Supporting Information). Mass spectrometry revealed that ME2 was strongly acetylated at K156 in cancer cells in response to cisplatin treatment (Figure 1E). Moreover, we verified ME2 K156 acetylation via a custom‐designed antibody. The specificity of the antibody for detecting ME2 K156 was validated by dot blotting (Figure 1F). The acetylation level of ME2 at K156 consistently and substantially increased in the acquired cisplatin‐resistant tumors (Figure 1G) and a panel of in vitro cisplatin‐induced ovarian cancer cell lines (Figure 1H; Figure S1A, Supporting Information). Given that the first‐line treatment for ovarian cancer patients is platinum‐based chemotherapy, we evaluated the clinical relevance of ME2 K156 acetylation in the context of chemotherapy resistance in tumor samples from ovarian cancer patients. We initially collected primary tumors from ovarian cancer patients who received mainly platinum‐based chemotherapy, including cisplatin or carboplatin, for the evaluation of ME2 ac‐K156 (Table S2, Supporting Information). Patients were stratified into two groups: the sensitive group (patients who had a durable response to platinum‐based chemotherapy for at least 6 months) and the resistant group (patients who relapsed within 6 months and did not respond to platinum therapy). A significantly greater level of ME2 ac‐K156 was detected in primary tumor samples from the resistant group than in those from the sensitive group (Figure 1I). In addition, a commercially available ovarian cancer tissue array containing primary tumor tissue from 128 ovarian cancer patients whose survival data were available was analyzed via immunohistochemistry (IHC) to evaluate ME2 ac‐K156 (Table S3, Supporting Information). Higher ME2 ac‐K156 expression in primary tumors was significantly associated with worse progression‐free survival (PFS) but not overall survival, suggesting that ME2 ac‐K156 may impede the response to platinum‐based chemotherapeutics (Figure 1J). Taken together, these data demonstrate that acetylation of ME2 at lysine 156 is associated with resistance to platinum‐based chemotherapy.

### 2.2 Acetylated ME2 Promotes the Survival and Growth of Cisplatin‐Resistant Cancer Cells

**Figure 2:**
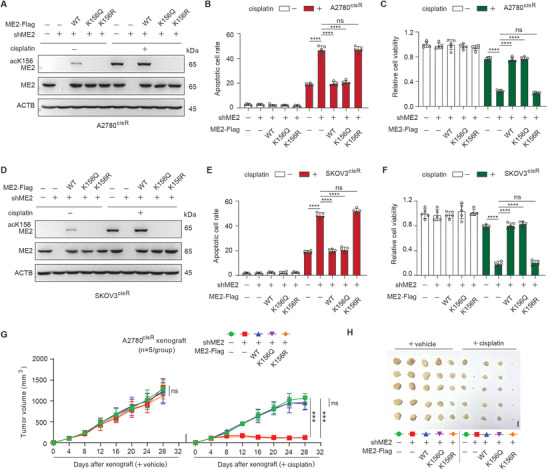
Acetylated ME2 promotes the survival and growth of cisplatin‐resistant cancer cells. A) Representative western blot of the indicated samples after treatment with cisplatin (5 µg ml^−1^, 48 h) using cisplatin‐resistant A2780 cells (A2780^cisR^). B) Apoptosis of the indicated samples was detected by flow cytometry after treatment with cisplatin (5 µg ml^−1^, 48 h). The data are presented as the means ± SDs (n = 3) and were analyzed by one‐way ANOVA. C) Cell viability of the indicated samples was detected via the trypan blue exclusion test in the presence of cisplatin (5 µg ml^−1^, 48 h). The data are presented as the means ± SDs (n = 5) and were analyzed by one‐way ANOVA. D) Representative western blot of the indicated samples after treatment with cisplatin (5 µg ml^−1^, 48 h) using cisplatin‐resistant SKOV3 cells (SKOV3^cisR^). E) Apoptosis of the indicated samples was detected by flow cytometry after treatment with cisplatin (5 µg ml^−1^, 48 h). The data are presented as the means ± SDs (n = 3) and were analyzed by one‐way ANOVA. F) Cell viability of the indicated samples was detected via the trypan blue exclusion test in the presence of cisplatin (5 µg ml^−1^, 48 h). The data are presented as the means ± SDs (n = 5) and were analyzed by one‐way ANOVA. (G‐H) Effect of the ME2 K156Q or K156R mutation on tumor growth under cisplatin treatment. The mice were treated with or without cisplatin (5 mg kg^−1^ i.p. twice per week) beginning at 4 days after xenograft, G) tumor growth curve (mean ± SD, 2‐way ANOVA), H) dissected tumors at the end point (scale bar, 10 mm). The data shown are representative of 2 independent biological experiments for (A) and (D). ***P < 0.001, ****P < 0.0001, ns: not significant. See also Figure S2 (Supporting Information).

To investigate the role of acetylated ME2 in cancer cell survival and growth in chemoresistance, cisplatin‐resistant ovarian cancer cell lines with stable knockdown of endogenous ME2 and forced expression of shRNA‐resistant wild‐type (WT), acetylation‐mimic (K156Q), or acetylation‐deficient (K156R) mutants of ME2 were generated (**Figure** **2**A,D) and assayed for cell survival and growth in vitro and in vivo. We found that ME2 depletion did not affect apoptosis induction or cell viability in the absence of cisplatin (Figure 2B,C,E,F). However, with cisplatin treatment, ME2 knockdown significantly increased apoptosis induction and decreased cell viability, whereas the expression of WT or K156Q, but not K156R, ME2 significantly rescued the phenotypes resulting from ME2 knockdown (Figure 2B,C,E,F). Moreover, similar effects were observed in the cisplatin‐resistant cell lines originating from both lung cancer (Figure S2A–C, Supporting Information) and head and neck cancer (Figure S2D–F, Supporting Information), implying a potential role of acetylated ME2 in chemotherapy resistance among various cancer types. Next, we validated the role of acetylated ME2 in vivo in a xenograft mouse model. We initially confirmed that tumors derived from A2780^cisR^ cells with ME2 knockdown exhibited apparent decreases in tumor growth and tumor size in mice treated with cisplatin (Figure S2G,H, Supporting Information). More importantly, we found that WT or K156Q ME2, not K156R ME2, restored the attenuated growth of ME2‐depleted A2780^cisR^ xenograft tumors treated with cisplatin (Figure 2G,H). These data reveal that acetylation of ME2 at K156 promotes cisplatin‐resistant cancer cell survival and tumor growth.

### 2.3 Acetylated ME2 Contributes to the Majority of Lactate Production via Glutaminolysis During Long‐Term Treatment with Cisplatin

**Figure 3:**
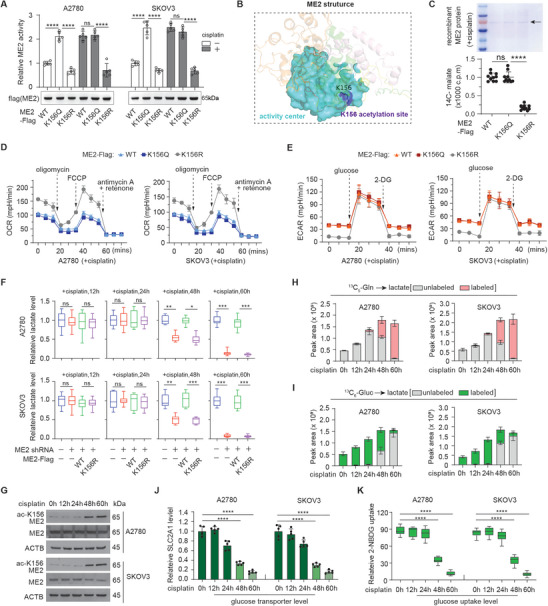
Acetylated ME2 contributes to the majority of lactate production via glutaminolysis over long‐term treatment with cisplatin. A) ME2 activity in the indicated samples treated with cisplatin (1 µg ml^−1^, 48 h). The data are presented as the means ± SDs (n = 5) and were analyzed by one‐way ANOVA. B) ME2 protein structure showing the activity center and K156 site. AlphaFoldDB: P23368. C) Binding affinity of ^14^C‐labeled malate for the recombinant ME2 protein determined by a scintillation counter. ME2 protein was purified from overexpressing 293T cells treated with cisplatin. The data are presented as the means ± SDs (n = 10) and were analyzed by one‐way ANOVA. D,E) Oxygen consumption rate (OCR) and extracellular acidification rate (ECAR) determined by the seahorse assay in the indicated cells co‐treated with cisplatin (1 µg ml^−1^), n = 3. F) Relative lactate levels in the indicated cells. The data are presented as box and whiskers (from min to max, n = 10) and were analyzed by one‐way ANOVA. cisplatin (1 µg ml^−1^). G) Western blot analysis of the indicated samples after treatment with cisplatin (1 µg ml^−1^). The blot shown is representative of 3 independent biological experiments. H,I) Lactate tracing via ^13^C_5_‐glutamine H) or ^13^C_6_‐glucose I) in the indicated samples; n = 3, cisplatin (1 µg ml^−1^). The peak area levels of lactate were normalized to the cell count. Unlabeled, M+0; labeled, M+1 to M+n. J) RT‐qPCR analysis of SLC2A1 level in the indicated cells co‐treated with cisplatin (1 µg ml^−1^). The data are presented as the means ± SDs (n = 5) and were analyzed by one‐way ANOVA. K) Glucose uptake was assessed using 2‐NBDG in indicated cells. The data are presented as box and whiskers (from min to max, n = 10) and were analyzed by one‐way ANOVA. ****P < 0.0001, ns: not significant. See also Figure S3 (Supporting Information).

We next explored how acetylated ME2 might affect the response of cancer cells to cisplatin. First, we investigated whether acetylation of ME2 at K156 is important for ME2 enzyme activity. We compared the enzyme activity of wild‐type (WT), acetylation‐mimetic (K156Q) or acetylation‐deficient (K156R) mutants of ME2 in cancer cells treated with or without cisplatin. In response to cisplatin, the enzyme activity of WT ME2 increased dramatically, reaching the level of K156Q ME2 (**Figure** **3**A). Protein structure analysis suggested that the K156 site is close to the substrate binding site of ME2 (Figure 3B). The in vitro binding assay further revealed that K156 acetylation significantly increased the affinity of ME2 for its substrate malate (Figure 3C). These data indicate that ME2 K156 acetylation enhances its enzyme activity in response to cisplatin treatment in cancer cells.

We then asked whether the upregulation of ME2 enzyme activity by ME2 K156 acetylation provides a metabolic advantage for cisplatin resistance. Given that ME2 is an enzyme located in the mitochondria, we assessed mitochondrial function in cancer cells expressing WT, K156Q, or K156R ME2 via the Seahorse assay (Figure 3D). Expression of WT or K156Q ME2 reduced the oxygen consumption rates in cisplatin‐treated cells, particularly the maximal respiration capacity, suggesting that acetylated ME2 could substantially switch the intermediate metabolite in the TCA cycle from energy production to biosynthesis via the conversion of malate to pyruvate. When oxidative respiration is limited, pyruvate is usually further converted into lactate. Indeed, as verified by the Seahorse assay, extracellular acidification rate (ECAR) increased markedly in WT or K156Q ME2‐expression cancer cells treated with cisplatin, particularly when glucose availability is inhibited (Figure 3E). Interestingly, in the cancer cells treated with cisplatin for extended time (48 and 60 h), ME2 knockdown significantly decreased lactate levels, whereas the expression of WT, but not K156R, ME2 significantly restored the lactate level resulting from ME2 knockdown (Figure 3F). Similar trend was observed in the cell viability, supporting the role of acetylated ME2‐mediated lactate production in chemoresistance (Figure S3A, Supporting Information). Interestingly, ME2 K156 acetylation appear not to involve in the regulation of lactate production in cancer cells under cisplatin treatment for shorter time (24 h or less), as the silencing of ME2 by shRNA did not decrease the lactate level during this period (Figure 3F). Besides, in the cancer cells without cisplatin treatment, glycolysis and lactate production seemed also not affected by ME2 K156 acetylation, as no difference was observed in both glycolysis and lactate production between WT, K156Q, or K156R ME2‐expressing cancer cells with endogenous ME2 depleted (Figure S3B,C, Supporting Information). Consistent with this observation, the K156 acetylation of ME2 increased markedly after an extended period of cisplatin treatment, ≈48 h after cisplatin treatment (Figure 3G). We further confirmed that lactate is mainly derived from glutamine but not glucose by tracing ^13^C_5_‐glutamine or ^13^C_6_‐glucose in cancer cells under long‐term cisplatin treatment. The labeling of lactate from ^13^C_6_‐glucose was strongly reduced in cancer cells beginning 48 h after cisplatin treatment (Figure 3H–J); in contrast, increased labeling of lactate from ^13^C_5_‐glutamine was identified 48 h post‐cisplatin, consistent with the role of acetylated ME2 and glutamine metabolism in supporting the production of lactate in the cancer cells over long‐term treatment with cisplatin. Furthermore, we observed cisplatin exposure suppressed expression of the major glucose transporter GLUT1 in cancer cells (Figure 3J; Figure S3D, Supporting Information). Glucose uptake was therefore compromised in cancer cells, and this defect was associated with treatment course (Figure 3K). In cancer cells beginning 48 h after cisplatin treatment, glucose transporter GLUT1 and glucose uptake decreased dramatically in cancer cells, which may force cells to rely on glutamine and acetylated ME2 for lactate production. Taken together, these data suggest that acetylated ME2‐mediated glutamine metabolism is possibly necessary for lactate production in cancer cells under long‐term chemotherapy treatment.

### 2.4 Acetylated ME2 is Important for Homologous Recombination Repair via Lactate

**Figure 4:**
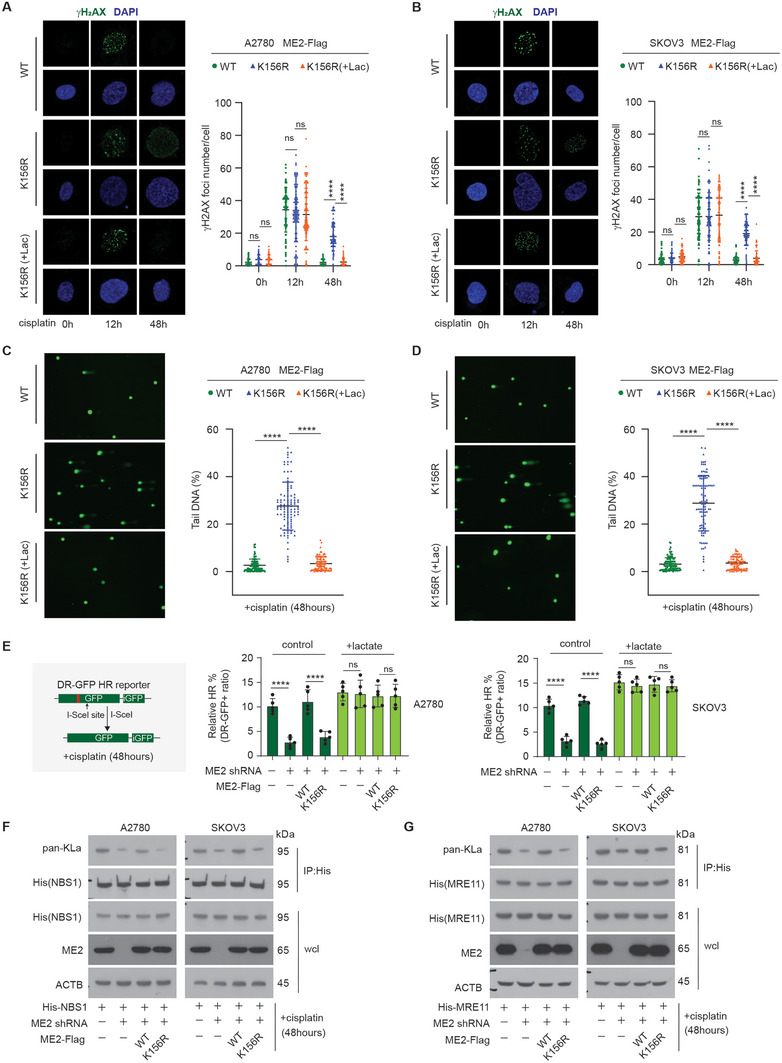
Acetylated ME2 is important for homologous recombination repair via lactate. A,B) Representative image (left) and quantification (right) of γH2AX foci in A2780 A) or SKOV3 B) cells treated as indicated. L‐lactate (20 mM) was added 24 h after cisplatin treatment (1 µg ml^−1^, 48 h). Scale bars, 10 µm. Two‐way ANOVA. C,D) Representative image (left) and quantification (right) of the comet assay results in A2780 A) or SKOV3 B) cells treated as indicated. L‐lactate (20 mM) was added 24 h after cisplatin treatment (1 µg ml^−1^). Scale bars, 10 µm. The proportion of DNA in the COMET “tail” was scored. One‐way ANOVA. E) DR‐GFP HR reporter assay was used to analyze HR efficiency in cells by GFP expression using flow cytometry. L‐lactate (20 mM) was added 24 h after cisplatin treatment (1 µg ml^−1^, 48 h). Two‐way ANOVA. (F‐G) Western blot analysis of the indicated samples after treatment with cisplatin (1 µg ml^−1^, 48 h). The blot shown is representative of 3 independent biological experiments. ****P < 0.0001, ns: not significant. See also Figure S4 (Supporting Information).

Lactate has been shown to increase the DNA damage repair capacity of cells under chemotherapy. We therefore considered whether our observations that acetylated ME2 promotes cisplatin‐resistant cancer cell survival and tumor growth might reflect an impact of ME2‐derived lactate on DNA damage repair. Compared with ME2‐depleted cells rescued with WT ME2, cells rescued with K156R ME2 presented significantly more cisplatin‐induced γH2AX foci (**Figure** **4**A,B) and γH2AX total level (Figure S4A, Supporting Information) at later time points after cisplatin treatment, confirmed by immunofluorescence and immunoblot, respectively, suggesting that acetylated ME2 indeed facilitated DNA damage repair. Moreover, lactate treatment completely reversed the phenotypes resulting from the ME2 K156R mutation, suggesting that acetylated ME2 works through lactate to facilitate DNA damage repair (Figure 4A,B). We further validated this observation via a comet assay to monitor DNA damage. Consistent with γH2AX, we observed a significantly greater amount of DNA in the comet tail in K156R ME2‐expressing cells than in WT ME2‐expressing cells 48 h after treatment with cisplatin (Figure 4C,D). In addition, lactate treatment markedly reduced the amount of DNA in the comet tail in K156R ME2‐expressing cells treated with cisplatin, further confirming that the axis of acetylated ME2‐lactate regulates DNA damage (Figure 4C,D).

Cancer cells rely mainly on homologous recombination (HR) or nonhomologous end joining (NHEJ) for DNA damage repair.^[^ ^15^ ^]^ We then examined whether acetylated ME2 is involved in the regulation of HR or NHEJ by performing I‐SecI‐based HR or NHEJ reporter assays in ME2‐deplated cells rescued with WT or K156R ME2 (Figure S4B, Supporting Information). The assays were performed in glucose‐free culture conditions to force reliance on glutaminolysis in cancer cells. We found that the knockdown of ME2 significantly suppressed HR repair, whereas the expression of WT, but not K156R, ME2 significantly rescued the phenotypes resulting from ME2 knockdown. Moreover, the impact of acetylated ME2 on HR repair was completely abrogated by L‐lactate treatment, suggesting that acetylated ME2 might regulate HR repair through lactate (Figure 4E). Unlike HR repair, acetylated ME2 and lactate treatment did not affect the level of NHEJ (Figure S4C, Supporting Information). Moreover, consistent with previous reports,^[^ ^2^, ^5^ ^]^ we found that key HR proteins, including MRE11 and NBS1 of the MRN complex, are strongly lactylated in cells treated with cisplatin for a long duration (48 h), which play critical roles in HR repair (Figure 4F). However, silencing ME2 with shRNA markedly reduced the lactylation levels of both MRE11 and NBS1, whereas the expression of WT, but not K156R, ME2 significantly restored the lactylation levels of both MRE11 and NBS1 (Figure 4G). These data reveal that acetylated ME2 is important for homologous recombination repair via lactate.

### 2.5 Acetyl Transferase ACAT1 and Deacetylase SIRT4 Counteract the Acetylation of ME2

**Figure 5:**
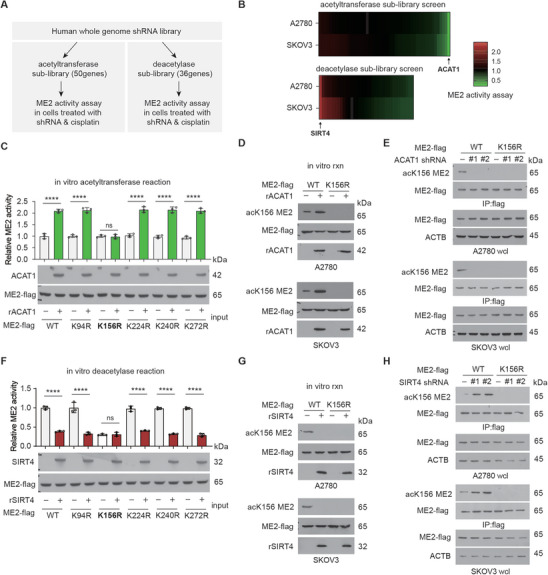
The acetyl transferase ACAT1 and deacetylase SIRT4 counteract the acetylation of ME2. A) Schematic representation of an ME2 activity assay‐based screening strategy to identify lead candidates as upstream acetyltransferases and deacetylases of ME2. cisplatin treatment (1 µg ml^−1^, 48 h). B) Heatmap showing the screening results of (A), n = 3. C) ME2 activity in the indicated samples from the in vitro acetyltransferase reaction, n = 3. A representative western blot showing the input of the indicated recombinant protein. D) Western blot of the indicated samples from the in vitro reaction using recombinant proteins. E) Western blot of the indicated samples from ME2‐flag WT‐ or K156R‐expressing cells with or without ACAT1 knockdown. F) ME2 activity in the indicated samples from the in vitro deacetylase reaction; n = 3. A representative western blot showing the input of the indicated recombinant protein. G) Western blot of the indicated samples from the in vitro reaction using recombinant proteins. H) Western blot of the indicated samples from ME2‐flag WT‐ or K156R‐expressing cells with or without SIRT4 knockdown. The blots shown are representative of 3 independent biological experiments. ****P < 0.0001, ns: not significant.

To identify upstream acetyltransferase(s) and deacetylase(s) of ME2 in cells under chemotherapy, we constructed two “targeted” lentiviral shRNA libraries that target 50 out of 71 acetyltransferases and 36 out of 40 deacetylases, respectively (Tables S5 and S6, Supporting Information), as previously described.^[^ ^19^ ^]^ Using these two libraries, we performed ME2 enzyme activity assay‐based screening studies in A2780 or SKOV3 ovarian cancer cells in which acetyltransferases or deacetylases were knocked down via lentiviral transduction followed by treatment with cisplatin (**Figure** **5**A). Candidates of ME2 acetyltransferase or deacetylase were ranked by the ability of the related shRNAs to abolish the cisplatin‐induced increase in ME2 activity or to further increase ME2 activity, respectively, upon lentiviral shRNA‐mediated knockdown and cisplatin treatment. We identified the mitochondrion‐localized enzyme acetyl‐CoA acetyltransferase 1 (ACAT1) as a potential ME2 acetyltransferase and SIRT4 as a potential ME2 deacetylase (Figure 5B). We next performed an in vitro acetyltransferase reaction using recombinant ME2 WT or diverse ME2 KR mutant proteins with the ACAT1 protein. Each of the recombinant ME2 WT or KR mutant proteins with low levels of lysine acetylation was enriched from overexpressing 293T cells with endogenous ACAT1 knockdown. We found that incubation with purified, recombinant ACAT1 resulted in increased enzyme activity of ME2 WT and various ME2 KR mutants but not the ME2 K156R mutant (Figure 5C). Western blot analysis also confirmed that such treatment strongly increased K156 acetylation in WT ME2 but not in the K156R mutant (Figure 5D). In cancer cells treated with cisplatin, ACAT1 knockdown strongly suppressed the K156 acetylation of WT ME2 (Figure 5E). These data suggest that ACAT1 is the upstream acetyltransferase for ME2 K156.

We also performed an in vitro deacetylase reaction using recombinant ME2 WT or diverse KR mutant proteins and the SIRT4 protein. We purified recombinant ME2 protein with high levels of lysine acetylation from overexpressing 293T cells treated with cisplatin and NAM+TSA. As shown in Figure 5F, incubation with purified, recombinant SIRT4 significantly inhibited the enzyme activity of WT ME2 and various ME2 KR mutants but not the ME2 K156R mutant (Figure 5F). Under these conditions, we also observed a significant decrease in K156 acetylation in ME2 WT in vitro, as shown by western blotting (Figure 5G). Moreover, SIRT4 knockdown potentiated the cisplatin‐induced increase in K156 acetylation in WT ME2 (Figure 5H). Together, these results suggest that ACAT1 and SIRT4 are upstream acetyltransferases and deacetylases that selectively acetylate and deacetylate K156 of ME2, respectively.

### 2.6 Decreased Pyruvate Production Resulting from Low Glucose Levels Promotes ACAT1 Binding to ME2 and Suppresses SIRT4 Binding to ME2

**Figure 6:**
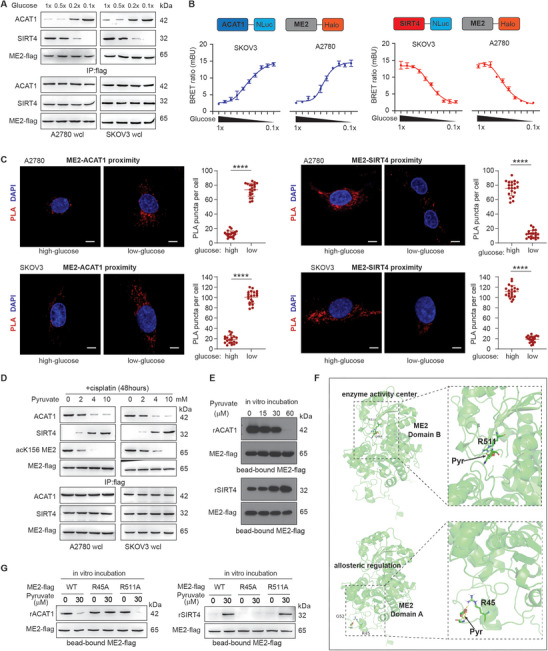
Decreased pyruvate production resulting from low glucose levels promotes ACAT1 binding to ME2 and suppresses SIRT4 binding to ME2. A) Western blot of the indicated samples from cells cultured in a series of decreasing concentrations of glucose. 1X: basic medium glucose level. B) BRET assay for the binding of ME2 with ACAT1 B) or SIRT4 C) measured in the indicated cells cultured in a series of decreasing concentrations of glucose. The data are presented as the means ± SDs; n = 3. C) Confocal analysis of in situ PLA using antibody against indicated protein in indicated cells cultured with high or low glucose. For each subpanel, left: representative confocal image, right: count of PLA puncta per cells in indicated group. The data are presented as the means ± SDs (n = 20) and were analyzed by one‐way ANOVA. Bar represents 10 µm. D) Western blot of the indicated samples from cells cultured with a series of concentrations of pyruvate and cotreated with cisplatin (1 µg ml^−1^, 48 h). E) Western blot of the indicated samples from the in vitro incubation assay using recombinant ACAT1 or SIRT4 and purified ME2‐flag protein in the presence of increasing concentrations of pyruvate. F) Molecular docking of pyruvate with ME2. ME2 proteins are shown as cartoons and colored light green. Pyruvate and critical residues are shown in stick representation. The top two binding modes are shown. G) Western blot of the indicated samples from the in vitro incubation assay using recombinant ACAT1 or SIRT4 and purified ME2‐flag WT or mutant protein in the absence or presence of pyruvate. The blots shown are representative of 3 independent biological experiments. ****P < 0.0001, ns: not significant. See also Figure S5 (Supporting Information).

We next investigated which mechanism determines the counteraction of acetyl transferase ACAT1 and deacetylase SIRT4 on the acetylation of ME2 at K156. The acetylation of ME2 at K156 increased dramatically in the later stages of cisplatin treatment, when influx of glucose is significantly inhibited, and the cells turned to glutaminolysis. We therefore hypothesized that low concentrations of glucose would stimulate the acetylation of ME2 and the conversion of glutamine to lactate to accelerate glutaminolysis, a process reminiscent of anaerobic glycolysis. To test this hypothesis, we examined the interaction of ME2 with ACAT1 or SIRT4 in cells cultured in a series of decreasing concentrations of glucose. Immunoprecipitation experiments demonstrated that as the concentration of glucose decreased, the interaction between ME2 and ACAT1 gradually increased, whereas the interaction between ME2 and SIRT4 gradually decreased (**Figure** **6**A). Moreover, decreasing glucose levels bona fide enhanced ACAT1 binding to ME2 but decreased SIRT4 binding to ME2 in intact cells, as indicated by the BRET assay in cancer cells co‐transfected with ME2‐Halo and ACAT1‐Nluc or with ME2‐Halo and SIRT4‐Nluc (Figure 6B) and by the proximity ligation assay (PLA) in cancer cells treated with high or low glucose (Figure 6C), respectively.

Our next question is how the mitochondrial protein interaction is regulated by the glucose concentration, especially considering that both glucose and glucose‐6‐phoshphate cannot enter the mitochondria. In fact, the common metabolite generated from glycolysis that can enter the mitochondria is pyruvate. Pyruvate itself is also the enzyme product of ME2. We found that pyruvate supplementation in the cell culture significantly inhibited ME2 K156 acetylation in the cisplatin‐treated cells in a dose‐dependent manner, which coincided with decreased ACAT1 binding and increased SIRT4 binding to ME2 (Figure 6D).

We then investigated whether pyruvate affects the interaction of ME2 with ACAT1 or SIRT4 via in vitro incubation. Flag‐beads bound ME2‐flag recombinant protein was incubated with recombinant ACAT1 or SIRT4 in the presence of increasing concentrations of pyruvate for 20 min at room temperature before the unbound protein was washed out. Western blot analysis revealed that pyruvate incubation directly decreased ME2 binding to ACAT1 while increasing ME2 binding to SIRT4 (Figure 6E). Protein‐metabolite docking simulations revealed that pyruvate could interact with ME2 both at the substrate binding sites of the enzyme activity center and at the allosteric regulation domain with high affinity, respectively (Figure 6F). Therefore, we generated potential pyruvate binding‐deficient mutants of ME2, including single‐mutant ME2 R511A (mutation of the predicted binding site in the enzyme activity center) or ME2 R45A (mutation of the predicted binding site in the allosteric regulation domain) and the double‐mutant ME2 R511A/R45A, and found that 14C‐labeled pyruvate bound to ME2 WT proteins with a KD value of ≈6.169 µM. Double mutation significantly decreased the binding of pyruvate to the ME2 protein (Figure S5A, Supporting Information). However, in vitro recombinant protein incubation revealed that pyruvate directly affected the in vitro interaction between ME2 and ACAT1/SIRT4, which was abolished by the ME2 R45A mutation but not the R511A mutation, confirming that pyruvate binding to ME2 in the allosteric regulation domain regulated the interaction between ME2 and ACAT1/SIRT4 (Figure 6G). Consistently, in cells cultured with cisplatin for longer durations (48 h), the binding of ME2 to ACAT1 increased and the binding of ME2 to SIRT4 decreased, which was abolished by the R45A mutation of ME2 (Figure S5B, Supporting Information). Taken together, these data suggest that decreased pyruvate production resulting from low glucose levels could promote ACAT1 binding to ME2 and suppress SIRT4 binding to ME2, thereby regulating the acetylation of ME2.

### 2.7 Pharmacological Inhibition of ACAT1 Inhibits ME2 Acetylation and Sensitizes Cancer Cells to Cisplatin Treatment

**Figure 7:**
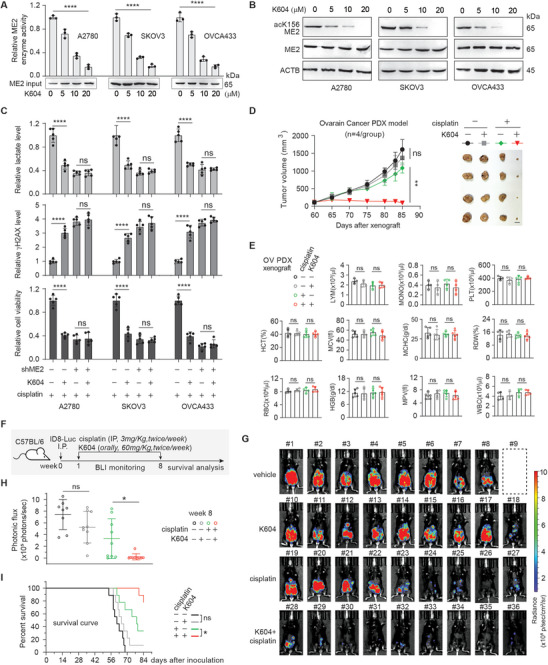
Pharmacological inhibition of ACAT1 inhibited ME2 acetylation and sensitized cancer cells to cisplatin treatment. A) ME2 activity in the indicated cells treated with cisplatin (1 µg ml^−1^, 48 h) and the ACAT1 inhibitor K604. Equal input control of ME2 protein was confirmed by western blot. The data are presented as the means ± SDs; n = 3; one‐way ANOVA. B) Western blot of the indicated samples from cells treated with cisplatin (1 µg ml^−1^, 48 h) and the ACAT1 inhibitor K604. The blots shown are representative of 3 independent biological experiments. C) Relative lactate levels, γH2AX levels and cell viability in cisplatin‐treated cells with or without ME2 knockdown in the absence or presence of K604. The data are presented as the means ± SDs; n = 5; one‐way ANOVA. D) Effect of K604 on the tumor growth of ovarian cancer PDX mice receiving cisplatin treatment. Cisplatin (5 mg kg^−1^ i.p., twice/week) and K604 (60 mg kg^−1^, twice per week) were administered. Tumor growth curves (left) and harvested tumors (right). Scale bars represent 10 mm for tumor images. The data are presented as the means ± SDs; n = 4; two‐way ANOVA. E) Hematology blood tests of the mice in the indicated groups. The data are presented as the means ± SDs; n = 4; one‐way ANOVA. F) Schematic illustration of the ID8 experimental design. G,H) Effects of combination treatment with K604 and cisplatin on ID8 tumor growth. Weekly average photonic flux G) and a representative bioluminescent image H) are shown. Cisplatin, 3 mg kg^−1^, i.p., twice per week; K604, 60 mg kg^−1^, twice per week. The data are presented as the means ± SDs; n = 5; two‐way ANOVA. I) Survival curves of ID8 tumor‐bearing mice. Log‐rank (Mantel‒Cox) test. ****P < 0.0001, ns: not significant.

Our finding that ME2 is acetylated by ACAT1 in tumors with low intracellular glucose to drive platinum resistance indicates that targeting ME2 acetylation could be a promising strategy to overcome platinum resistance. Therefore, we tested the efficacy of pharmacologically targeting the ACAT1‐ME2 axis on the cisplatin response in cancer cell lines and mouse tumor models. A selective ACAT‐1 inhibitor, K‐604,^[^ ^20^ ^]^ effectively inhibited ME2 enzyme activity and ME2 K156 acetylation in cancer cells treated with cisplatin (**Figure** **7**A,B). Inhibition of the ACAT1‐ME2 axis by K‐604 treatment resulted in decreased lactate levels, up‐regulated γH2AX staining, and increased cisplatin response in cancer cells. The K‐604 effect was abolished in ME2‐depleted cells (Figure 7C). Finally, we evaluated the effects of pharmacological inhibition of ACAT1 on cisplatin‐resistant tumor growth in vivo in PDX and ID8 peritoneal tumor models of ovarian cancer. Compared with cisplatin treatment alone, the combination of K‐604 and cisplatin significantly reduced tumor growth and tumor size in ovarian cancer PDXs (Figure 7D). The dosages of 60 mg kg^−1^ K‐604 and 5 mg kg^−1^ cisplatin did not induce significant changes in hematopoietic properties (Figure 7E). In the ID8‐Luc syngeneic ovarian cancer mouse model (Figure 7F), tumor growth was monitored via in vivo bioluminescence imaging (BLI). The combination treatment of K‐604 and cisplatin markedly decreased the tumor growth potential, prolonged the response time to cisplatin (Figure 7G,H) and improved the survival of the mice (Figure 7I), whereas no significant difference in tumor growth was observed in control mice treated with either the vehicle control or K‐604 alone. These data provide evidence that targeting the ACAT1‐ME2 axis could serve as an effective strategy to overcome resistance to platinum‐based chemotherapy in cancer.

## 3 Discussion

Here, we identified ME2 as a critical regulator of lactate production that plays an important role in the lactylation of DNA repair proteins and acquired chemoresistance in cancer cells. An increasing number of studies have shown that lactate and lactylation play important roles in various pathophysiological conditions.^[^ ^21^ ^]^ Our findings suggest a mechanistic basis by which cancer cells maintain levels of lactate from glutaminolysis via the acylation of ME2. Aerobic glycolysis, or the Warburg effect, predominantly contributes to lactate production when the glucose supply is sufficient. Consistently, although ME2 is expressed in cells, cancer cells rely on aerobic glycolysis to produce lactate when glucose levels are high. However, when cancer cells are treated with cisplatin for extended periods of time to acquire chemoresistance, intracellular glucose levels decrease significantly due to inhibition of the influx of glucose. The declining glucose level promotes the acetylation of ME2 by increasing the binding of acetyltransferase ACAT1 to ME2 and decreasing the binding of the deacetylase SIRT4 to ME2. Acetylation of ME2 induced by low intracellular glucose significantly increases its enzymatic activity to produce pyruvate and its derivative lactate from glutamine‐derived malate. ME2‐mediated lactate production from glutamine is important for the lactylation of DNA repair proteins when the glucose supply is low. Our studies using clinical samples support that upregulation of ME2 acetylation is positively correlated with nonresponse to platinum therapy and decreased progression‐free survival in ovarian cancer patients who have received chemotherapy as first‐line treatment.

The results of the present study demonstrated that ME2 is significantly acetylated at the K156 site to increase its enzyme activity and confer chemoresistance. Posttranscriptional modifications of proteins have long been shown to play important roles in chemotherapy resistance. We and others have shown that MAST1‐mediated phosphorylation of MEK1,^[^ ^10^, ^18^ ^]^ redox‐regulated phosphorylation of ITPKB,^[^ ^22^ ^]^ and acetylation of KLF5^[^ ^23^ ^]^ play critical roles in chemoresistance by regulating the cell survival signaling pathway, redox balance and epithelial‒mesenchymal transition (EMT), respectively. Moreover, histone modification plays a direct role in regulating DNA repair in response to DNA damage. We revealed that K156 acetylation of ME2 significantly increases its enzyme activity and thereby converts malate, a metabolite in the TCA cycle, to pyruvate, the substrate of lactate dehydrogenase (LDH). K156 acetylation of ME2 may induce conformational changes that significantly increase its binding affinity for malate; therefore, malate is less likely to be catalyzed by malate dehydrogenase to produce oxaloacetate in the TCA cycle. Since glutaminolysis replenishes the intermediate of the TCA cycle when glucose is low, K156 acetylation of ME2 links glutaminolysis to lactate production. While the K156 site is close to the enzyme activity center of ME2, future mutational and structural studies are warranted to determine the structural mechanisms by which the acetylation of K156 increases its binding affinity for malate and enzyme activity.

Since the acetylation of ME2 occurs when glucose levels decrease significantly, a cellular event related to glycolysis may directly affect the acetylation status of ME2. Using the shRNA‐based screen we previously described,^[^ ^19^, ^24^ ^]^ we first identified that ACAT1 and SIRT4 directly acetylate and deacetylate ME2, respectively. Intriguingly, we revealed that pyruvate, the final product of glycolysis, directly binds to ME2 in the allosteric regulation domain. Moreover, this allosteric regulation by pyruvate suppresses the ACAT1‐mediated acetylation of ME2 but promotes the SIRT4‐mediated deacetylation of ME2, leading to a decrease in the overall acetylation level and enzyme activity of ME2. As the metabolic product of ME2, pyruvate can also bind to the activity center of ME2 and directly inhibit its activity in a feedback manner. Therefore, our study reveals a dual regulatory mechanism of ME2 by pyruvate. Future studies that determine the complete cocrystal structure of the ME2/pyruvate complex may reveal the atomic details that affect the interaction of ACAT1/SIRT4 and ME2.

From a clinical perspective, our findings support the idea that the inhibition of ME2 acetylation could be a promising therapeutic strategy to treat cancer patients in combination with platinum‐based chemotherapy. We found that ME2 K156 acetylation confers platinum resistance in vivo and in vitro and that the acetylation of ME2 by ACAT1 is important for lactate production and DNA damage repair via the lactylation of DNA repair proteins. Moreover, we identified the ACAT1 inhibitor K‐604 as a potent ME2 K156 acetylation inhibitor with promising potential to overcome chemoresistance. These findings provide evidence that ACAT1 could be a direct target to inhibit ME2 K156 acetylation for therapeutic intervention in combination with cisplatin‐based chemotherapy. Notably, ACAT1 has important functions in ketogenesis and ketolysis by catalyzing the reversible formation of acetoacetyl‐CoA from two molecules of acetyl‐CoA.^[^ ^25^ ^]^ Moreover, ACAT1 also possesses acetyltransferase activity capable of acetylating pyruvate dehydrogenase (PDH). ACAT1‐dependent acetylation of PDH inhibits the PDH enzyme to increase pyruvate levels, thereby enhancing the Warburg effect and tumor growth.^[^ ^26^ ^]^ Consistent with this finding, our study demonstrated that ACAT1 could also increase pyruvate and lactate levels via direct acetylation of ME2, reinforcing the role of ACAT1 in cancer progression. Therefore, targeting ACAT1 could be a promising therapeutic strategy to overcome resistance to platinum‐based chemotherapy in future cancer treatment.

## 4 Experimental Section

### Cell Lines

A2780 and A549 cells (RPMI‐1640 medium supplemented with 10% fetal bovine serum (FBS) and 1% penicillin/streptomycin); OVCA433, 293T and PCI‐37B cells (DMEM supplemented with 10% FBS and 1% penicillin/streptomycin); SKOV3 cells (McCoy's 5a medium supplemented with 10% FBS and 1% penicillin/streptomycin); and ID8 cells cultured in complete DMEM supplemented with 10% FBS and insulin‐transferrin‐selenium‐ethanolamine (1%). SKOV3, 293T, and A549 cells were purchased from the American Type Culture Collection. A2780 and ID8 cells were obtained from Sigma Aldrich. PCI‐37B cells were obtained as previously described and the cisplatin‐resistant cell lines were generated from the parental cells after they were cultured with increasing doses of cisplatin, as previously described.^[^ ^22^ ^]^ All the cell lines were authenticated via STR profiling and tested for mycoplasma contamination.

### Reagents

All key reagents used in this study were listed in Table S4 (Supporting Information). All unique/stable reagents generated in this study were available from the corresponding author with a completed Materials Transfer Agreement.

### Proteome‐Wide Acetylation Analysis

Fresh tumor tissue was cut into ≈2 mm sections and crushed with the barrel of a syringe to form a homogenate. The cell homogenate was digested with RPMI 1640 medium containing Type II collagenase (2.5 mg mL^−1^, Thermo Fisher), 1× GlutaMAX (Thermo Fisher), 1% HEPES and 1% penicillin–streptomycin in a 37 °C water bath for 25 min, filtered through a 70 µm filter and centrifuged at 300 × g to pellet the cells. The cell samples were sonicated three times on ice via a high‐intensity ultrasonic processor (Scientz) in lysis buffer (8 M urea, 1% protease inhibitor cocktail, 3 µM TSA and 50 mM NAM). The remaining debris was removed by centrifugation at 12 000 × g at 4 °C for 10 min. Finally, the supernatant was collected, and the protein concentration was determined with a BCA kit according to the manufacturer's instructions. For trypsin digestion, the protein mixture was reduced with 5 mM dithiothreitol for 30 min at 56 °C and alkylated with 11 mM iodoacetamide for 15 min at room temperature in darkness. The protein sample was then diluted by adding 100 mM TEAB to a urea concentration of less than 2 M. Finally, trypsin was added at a 1:50 trypsin‐to‐protein mass ratio for the first digestion overnight and a 1:100 trypsin‐to‐protein mass ratio for a second 4 h digestion. Finally, the peptides were desalted on a C18 SPE column. To enrich the acetylated peptides, tryptic peptides dissolved in NETN buffer (100 mM NaCl, 1 mM EDTA, 50 mM Tris‐HCl, 0.5% NP‐40, pH 8.0) were incubated with prewashed anti‐acetyllysine antibody‐conjugated agarose beads (PTM‐104, PTM Bio) at 4 °C overnight with gentle shaking. The beads were subsequently washed four times with NETN buffer and twice with H_2_O. The bound peptides were eluted from the beads with 0.1% trifluoroacetic acid. Finally, the eluted fractions were combined and vacuum dried. The resulting peptides were desalted with C18 ZipTips (Millipore) for LC‒MS/MS analysis according to the manufacturer's instructions. The detailed results of the proteome‐wide acetylation mass spectrometry analysis were shown in Table S1 (Supporting Information).

### Immunohistochemistry

Resected tumors were fixed in 10% buffered formalin, embedded in paraffin and mounted on slides. After deparaffinization and rehydration, tumor section slides were incubated in 3% hydrogen peroxide to suppress endogenous peroxidase activity. Antigen retrieval was achieved by microwaving the sections in 10 mM sodium citrate (pH 6.0). Sections were then blocked by incubation in 2.5% horse serum containing avidin and biotin. The primary antibody (Invitrogen) was applied overnight at 4 °C. Detection was achieved with an avidin‐biotin complex system (Vector Laboratories). The slides were stained with 3,3′‐diaminobenzidine, washed, counterstained with hematoxylin, dehydrated, treated with xylene, and mounted. Tissue samples for the immunohistochemistry study were obtained with protocols reviewed and approved by the Institutional Review Board at Sun Yat‐sen University. The study was conducted in compliance with all ethical standards and good clinical practice. All clinical samples were collected with written informed consent from the participants or their guardians. Patient information was listed in Tables S2 and S3 (Supporting Information).

### Vector Constructs

The Gateway cloning strategy was used for the construction of the indicated vectors. The specific ORFs of the indicated genes were tagged or nontagged via PCR amplification via Q5 high‐fidelity DNA polymerase and subsequently cloned and inserted into pENTR/D‐TOPO, which was subsequently subcloned and inserted into the pLHCX‐derived Gateway destination vector for overexpression in mammalian cells or into pET‐53‐DEST (N‐terminal His tag) and pET‐60‐DEST (N‐terminal GST tag) for protein overexpression/purification in bacteria via LR Clonase II (Thermo Fisher Scientific).

### Cell Apoptosis Assay

Cell apoptosis was assessed via a FITC Annexin V apoptosis detection kit (BD Biosciences) according to the manufacturer's instructions.

### Cell Viability Assays

The viability of the indicated cell samples was determined via the fully automated trypan blue dye exclusion method via a cell counter (Beckman Coulter).

### ME2 Activity Assays

ME2 enzyme activity was measured as previously described.^[^ ^27^ ^]^ Briefly, the indicated cells were lysed in cell fraction buffer (20 mM HEPES‐KOH, pH 7.5, 10 mM KCl, 1.5 mM MgCl_2_, 1 mM sodium EDTA buffer, 1 mM sodium EGTA buffer, 1 mM dithiothreitol, and protease inhibitor cocktail) in the presence of 250 mM sucrose. The mitochondrial fraction or purified ME2 protein concentration was determined by BCA, and protein samples were added to an enzyme mixture containing 50 mM Tris‐HCl (pH 7.4), 10 mM MgCl_2_, 0.3 mM NAD+, and 3.3 mM L‐malic acid. The reactions were measured by absorbance at 340 nm every 20 s for up to 10 min. A mixture without L‐malic acid was used as a background control. The enzymatic activity was determined by subtracting the activity of the background control from that of each sample. The absorbance changes were normalized to the protein concentration.

### Lactate Level Assays and Lactate Tracing by MassSpec

Cellular lactate production was measured under normoxia with a lactate assay kit (ab65331, Abcam). Carbon incorporation into lactate from glutamine was determined by stable isotope tracing. Glutamine labeled with ^13^C was obtained from Sigma‒Aldrich (Cat#:605 166, L‐glutamine‐^13^C_5_). The medium for the cells cultured under the indicated conditions as described in the figure legends was replaced with RPMI‐1640 containing labeled nutrients. For glutamine tracing, the media were replaced with glutamine‐free RPMI‐1640 supplemented with 2.1 mM ^13^C_5_‐glutamine and 10% dialyzed FBS for 4 h before metabolite extraction or before cell fractionation followed by metabolite extraction. The cell number for each condition was determined by setting up parallel wells and used for normalization. The subsequent GC‒MS analysis was performed as previously described.^[^ ^15^ ^]^

### Immunofluorescence Assay

One milliliter of cells (1 × 10^5^ mL^−1^) was seeded onto a microscope cover glass in 12‐well cell culture plates overnight and then treated with the indicated concentration. After treatment, the cells were fixed with 4% paraformaldehyde, permeabilized with 0.5% Triton X‐100, blocked with normal goat serum, and incubated with Alexa Fluor 488–conjugated anti–p–histone H2A. X (Ser139) antibody (05‐636‐AF488; Millipore Sigma) for 1 h at room temperature. Nuclei were counterstained with DAPI (1 mg mL^−1^). Images were obtained via a Zeiss confocal microscope.

### Comet Assay

A comet assay kit (catalog 4250‐050‐K; Trevigen) was used to perform the alkaline comet assay according to the manufacturer's instructions. Briefly, a 5 µL volume of cells at 1 × 10^5^ mL^−1^ was added to 50 µL of molten LMA agarose (at 37 °C), and 50 µL of mixture was immediately pipetted onto a comet slide. After the mixture of agarose and cells was evenly dispersed, the slides were placed flat at 4 °C in the dark for 30 min in a high‐humidity environment. The cells were then lysed overnight by immersing the slides in lysis buffer. After lysis, the excess buffer was removed from the slides, and the slides were immersed in freshly prepared alkaline unwinding solution, pH >13, for 1 h at 4 °C in the dark before application of an electric field. The electrophoresis was performed under ice. An electric field (typically 1 V cm^−1^) was applied to the cells for 45 min at 4 °C, and the cells were stained with SYBR Gold (catalog S11494; Thermo Fisher Scientific) for 30 min in the dark and photographed via a Zeiss microscope with an attached camera. The comets were analyzed via ImageJ.

### Western Blotting

Total cellular protein was extracted from fresh cell cultures or tissues with lysis buffer (50 mM Tris‐HCl (pH 7.5), 150 mM NaCl, 1% NP‐40, and 0.5% Na‐deoxycholate supplemented with protease inhibitor cocktail (Roche)). SDS‒PAGE was performed to resolve a total of 30 µg of protein, which was subsequently transblotted onto a nitrocellulose membrane (Bio‐Rad). The membranes were probed with the indicated primary antibodies at 4 °C overnight, followed by incubation with HRP‐conjugated secondary antibodies for 1 h at room temperature. ECL was used to detect specific blot bands. Blot bands were quantified by ImageJ. Primary antibodies against the following proteins were used: ME2 (Cat# 12 399), ACTB (Cat# 12 620), Flag‐Tag (Cat# 5407), His‐Tag (Cat# 2365), MRE11 (Cat# 4895), RAD50 (Cat# 3427), NBS1 (Cat# 14 956), Histone H3 (Cat# 9733), ACAT1 (Cat# 44 276), and γH2AX (Cat# 20 304 were purchased from Cell Signaling Technology; Pan‐L‐Lactyllysine (PTM‐1401) was purchased from PTM‐BIO; SIRT4 Cat# ab105039) was purchased from Abcam; and ME2 ack 156 was generated by immunization of rabbits at ABclonal.

### RNAi Screen for Upstream Acetyltransferase and Deacetylase of ME2

Primary screening was performed via a lentiviral shRNA library of known acetyltransferases and deacetylases sorted from the TRC Lentiviral shRNA Library obtained from Open Biosystems. A detailed list of genes and target sequences were provided in Tables S5 and S6 (Supporting Information). Each acetyltransferase or deacetylase gene was targeted by a short hairpin RNA (shRNA) pool that contains two to five different lentiviral‐based shRNA constructs that target different regions of the target gene. A ME2 enzyme assay‐based screening strategy was designed to identify upstream acetyltransferases and deacetylases of ME2 via these shRNA pools. In brief, A2780 or SKOV3 ovarian cancer cells were infected with lentiviruses targeting each acetyltransferase or deacetylase. Four days after lentiviral infection, the cells were treated with cisplatin (1 µg ml^−1^) for 48 h and then harvested for the ME2 activity assay.

### 14C‐Pyruvate and ME2 Binding Assay

C‐terminal Flag‐tagged ME2 recombinant protein variants were prebound with Flag beads and then incubated with the indicated concentrations of 14C‐labeled pyruvate in 1x TBS buffer (50 mM Tris, 150 mM NaCl, pH 7.5) for 4 h at 4 °C. The beads were then washed three times with TBS buffer at 4 °C. and eluted by incubation with 10 mg of 3x Flag peptide (Sigma‒Aldrich) for 1 h. The supernatant containing the eluted ME2 protein was transferred to a scintillation vial containing liquid scintillation cocktails and immediately subjected to scintillation counting. Kd values were calculated via GraphPad Prism software.

### BRET Assay

ACAT1 and SIRT4 were cloned and inserted into vectors with a C‐terminal Nluc fusion tag, and ME2 was cloned and inserted into vectors with a C‐terminal Halo fusion tag via the Flexi Vector Cloning System (Promega). The indicated cells cultured with different concentrations of glucose in the medium were transfected with the combination of BRET plasmids for 12 h and then harvested to measure the bioluminescence according to the manufacturer's instructions (catalog N1821; Promega).

### In Vivo Study

Animal studies were performed according to protocols reviewed and approved by the Institutional Animal Care and Use Committee of Sun Yat‐sen University. Nude mice (Hsd: Athymic Nude‐Foxn1^nu^, female, 6‐week‐old, Envigo) were injected with 0.5 × 10^6^ KB‐3‐1 cells. For the in vivo induction model of cisplatin‐resistant cancer, nude mice bearing A2780 xenografts were administered a subeffective dose (0.8 mg kg^−1^) or a relatively high dose (6 mg kg^−1^) on days 4 and 10, respectively. Tumors were harvested at indicated endpoints. The cancer cells were selectively separated from the cell mixture using the Tumor Cell Isolation Kit for human cells (Product Number: 130–108–339; supplied by Miltenyi Biotec), adhering to the protocols provided by the manufacturer. In summary, the process involved labeling non‐tumor cells, such as various lymphocyte subsets, fibroblasts, and endothelial cells, with a mixture of monoclonal antibodies coupled to microbeads. These labeled cells were then effectively removed from the tumor cells by applying a magnetic field in a separation device. The purified cancer cells obtained were subsequently employed for determining the half‐maximal inhibitory concentration (IC50) of cisplatin. For ME2 functional studies, nude mice bearing A2780^cisR^ xenografts were administered cisplatin (5 mg kg^−1^) two times a week by intraperitoneal (i.p.) injection when the tumor size reached 100 mm^3^ on day 4. The ovarian cancer PDX model was obtained from the Jackson Laboratory. Ovarian cancer patient tumors were implanted into NOD scid gamma mice (female, 6‐week‐old, Jackson Laboratory). Approximately 1500 mm^3^ sized tumors were excised, evenly diced, and implanted in the flanks of the nude mice. The mice were randomly divided into groups when the tumor size reached 100 mm^3^. For the ID8 syngeneic mouse model, luciferase‐expressing ID8 cells were injected into C57BL/6 mice (female, 6‐week‐old, Envigo) by intraperitoneal (i.p.) injection. Cisplatin (3 mg kg^−1^) was given by i.p. injection twice a week. K604 (60 mg kg day^−1^) was orally administered. The volume of each xenograft tumor was calculated as 4*π*/3 x (width/2)^2^ x (length/2). Blinding and allocation concealment were used. For the syngeneic ID8 mouse model, tumor growth was monitored via bioluminescence imaging (BLI) analysis as described previously. Blood was harvested at the experimental endpoint for a complete blood count (CBC) test via flow cytometry analysis.

### Statistical Analysis

GraphPad Prism 8 (GraphPad Software) was used to perform the statistical analysis. The data represent the means ± SDs. No data were excluded. Statistical analysis of significance was based on a 2‐tailed Student's t test or 1‐way or 2‐way ANOVA with Bonferroni's post hoc multiple‐comparison test. P values of 0.05 or less were considered statistically significant. Statistical analyses were based on a set of assumptions, such as homogeneity of variances and a normal distribution. The variance was similar between the groups that were statistically compared. No statistical method was used to predetermine sample size; a significant difference was detected in the preliminary studies in this assay, so we used the minimum sample size for all in vivo experiments. In vitro experiments were performed at least 3 times per standard practice.

## Conflict of Interest

The authors declare no conflict of interest.

## Supporting information



Supporting Information

## Data Availability

The data that support the findings of this study are available from the corresponding author upon reasonable request.
