# Supplementary material for: ACAT1‐Mediated ME2 Acetylation Drives Chemoresistance in Ovarian Cancer by Linking Glutaminolysis to Lactate Production
Source: Adv Sci (Weinh). 2025 Feb 14;12(14):2416467. doi: 10.1002/advs.202416467 (PMC11984883; doi:10.1002/advs.202416467)
Supplement: Supplementary file 1 — Supporting Information [file ADVS-12-2416467-s001.docx]

­Supporting Information

**ACAT1-mediated ME2 acetylation drives chemoresistance in ovarian cancer by linking glutaminolysis to lactate production**

*Cuimiao Zheng, Hao Tan, Gang Niu, Xi Huang, Jingyi Lu, Siqi Chen, Haoyuan Li, Jiayu Zhu, Zhou Zhou, Manman Xu, Chaoyun Pan, Junxiu Liu, and Jie Li**

**Figure S1**

**
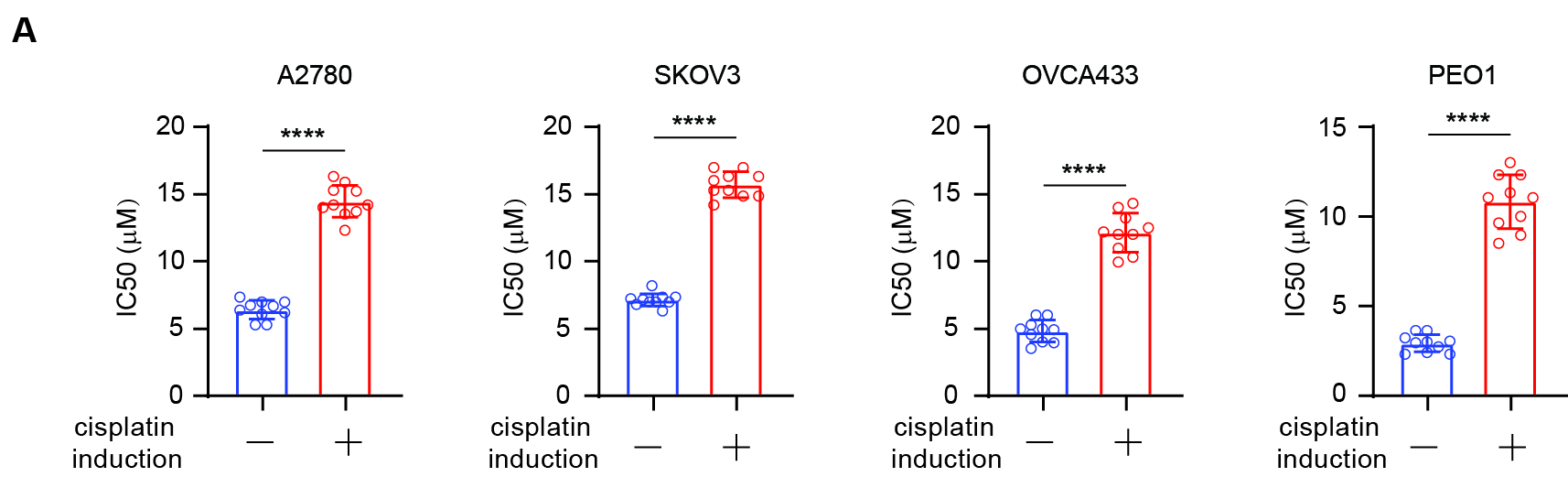
**

**Figure S1.** Related to **Figure 1**. Acetylation of ME2 at the lysine 156 site is associated with platinum resistance. (**A**) IC50 of cancer cells with or without cisplatin resistance induction. The data are presented as the means ± SDs (n=10) and were analyzed by2-tailed Student’s t test. ****P < 0.001.

**Figure S2**

**
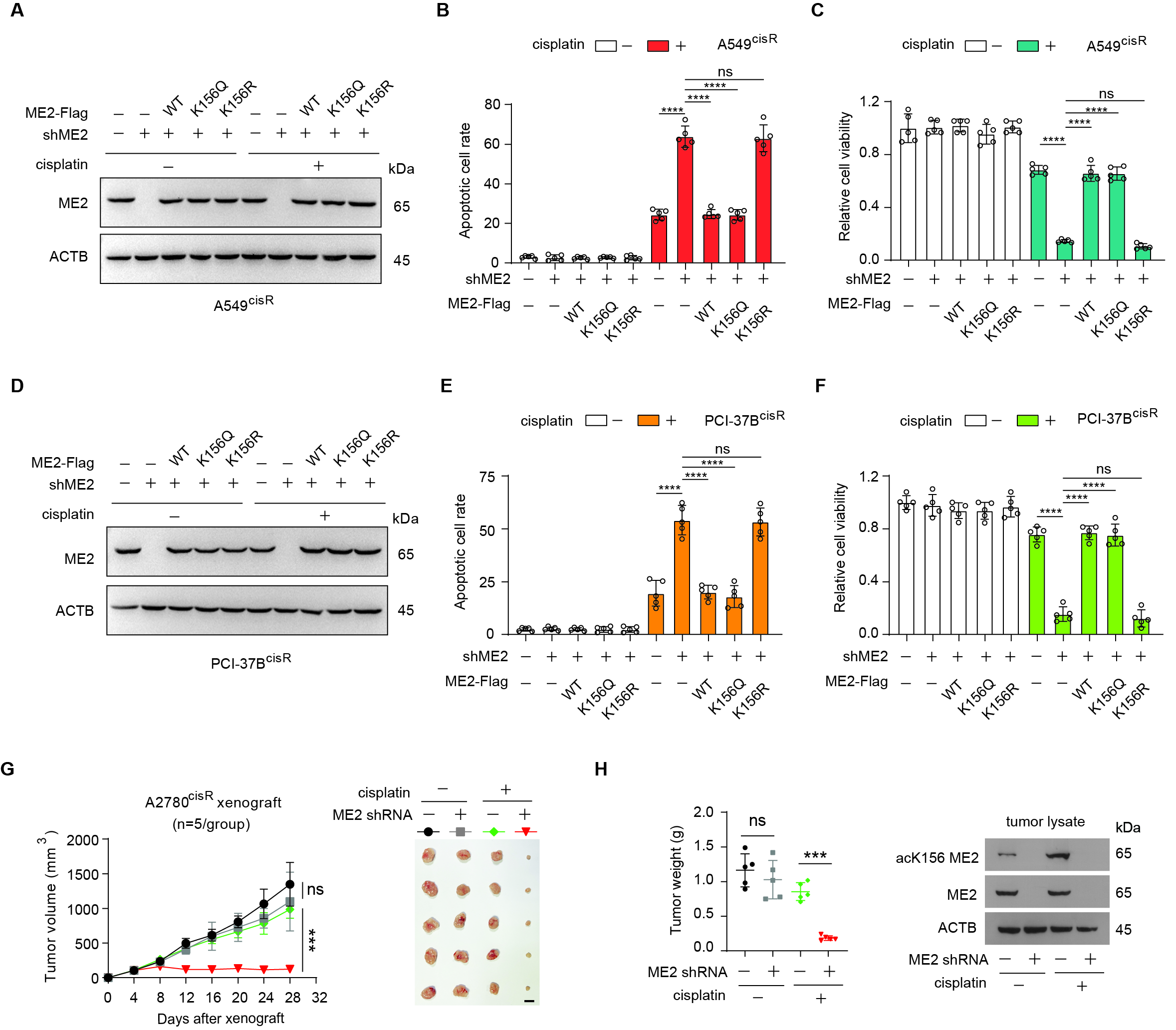
**

**Figure S2.** Related to Figure 2. Acetylated ME2 promotes the survival and growth of cisplatin-resistant cancer cells. (**A**) Representative western blot of the indicated samples after treatment with cisplatin (5 µg/ml, 48 hours) using cisplatin-resistant lung cancer cell line A549 cells (A549^cisR^). (**B**) Apoptosis of the indicated samples was detected by flow cytometry after treatment with cisplatin (5 µg/ml, 48 hours). The data are presented as the means ± SDs (n=3) and were analyzed by one-way ANOVA. (**C**) Cell viability of the indicated samples was detected via the trypan blue exclusion test in the presence of cisplatin (5 µg/ml, 48 hours). The data are presented as the means ± SDs (n=5) and were analyzed by one-way ANOVA. (**D**) Representative western blot of the indicated samples after treatment with cisplatin (5 µg/ml, 48 hours) using cisplatin-resistant head and neck cancer cell line (PCI-37B^cisR^). (**E**) Apoptosis of the indicated samples was detected by flow cytometry after treatment with cisplatin (5 µg/ml, 48 hours). The data are presented as the means ± SDs (n=3) and were analyzed by one-way ANOVA. (**F**) Cell viability of the indicated samples was detected via the trypan blue exclusion test in the presence of cisplatin (5 µg/ml, 48 hours). The data are presented as the means ± SDs (n=5) and were analyzed by one-way ANOVA. (**G-H**) Effects of ME2 knockdown and cisplatin treatment on tumor growth. The mice were treated with PBS or cisplatin (5 mg/kg i.p. twice per week) beginning at 4 days after xenograft. (A) left panel: tumor growth curve (mean ± SD, 2-way ANOVA), (A) right panel: dissected tumors at the end point (scale bar, 10 mm); (B) tumor weight at the end point (mean ± SD, 1-way ANOVA) and ME2 acK156 level in the harvested tumor lysate. ***P < 0.001, ns: not significant.

**Figure S3**


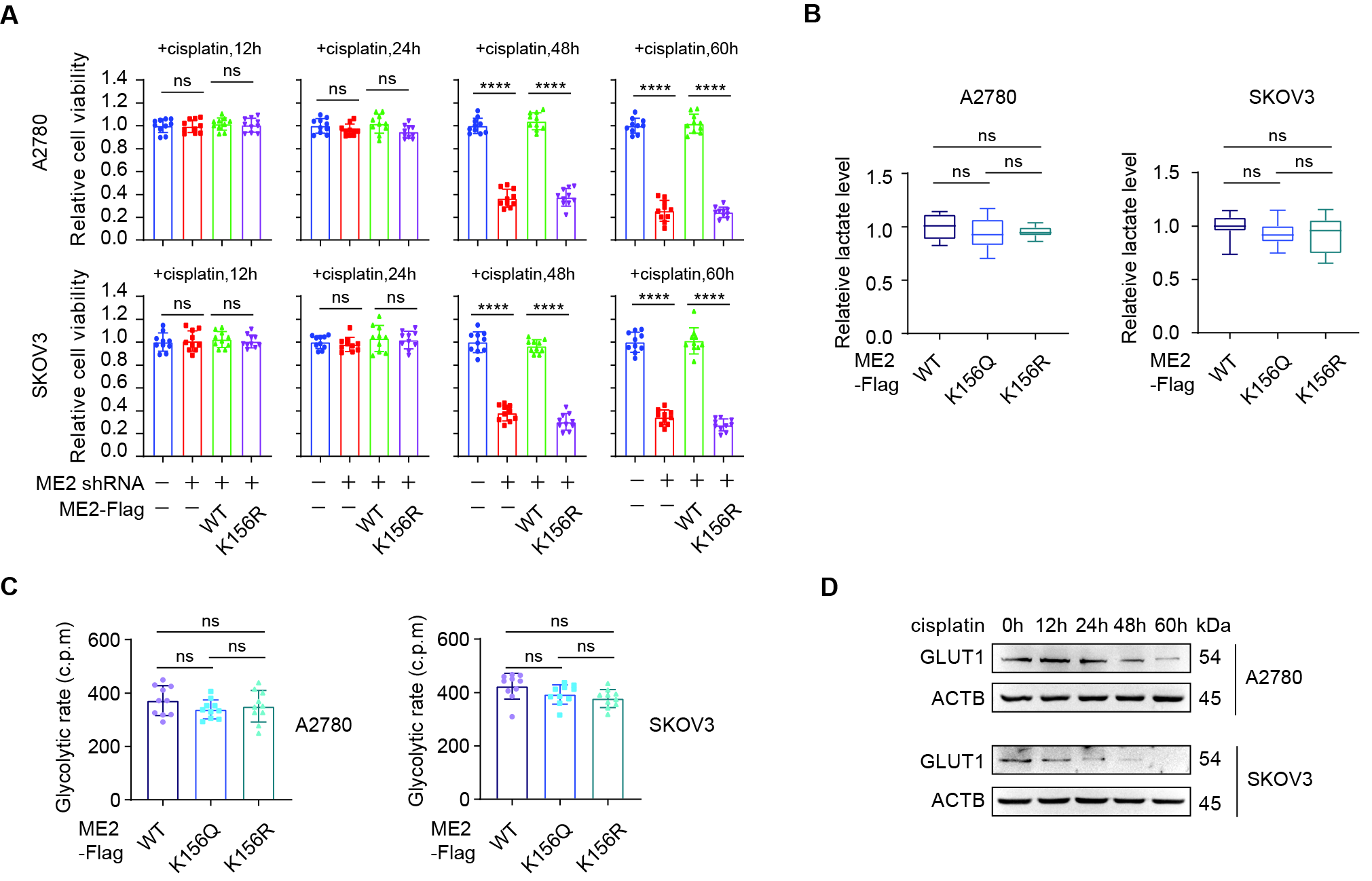


**Figure S3.** Related to Figure 3. Acetylated ME2 contributes to the majority of lactate production via glutaminolysis over long-term treatment with cisplatin. (**A**) Cell viability of the indicated cells treated with cisplatin (1 µg/ml). The data are presented as the means ± SDs (n=5) and were analyzed by one-way ANOVA. (**B**) Relative lactate levels in the indicated cells. The data are presented as box and whiskers (from min to max, n=10) and were analyzed by one-way ANOVA. (**C**) Glycolytic rates were measured by ^3^H_2_O release. The indicated cancer cells were spiked with 20 μCi/ml of D-[U-^3^H]-glucose in Krebs buffer supplemented with 10 mM glucose for 1 hour. Diffused ^3^H2O from d-[U-^3^H]-glucose was measured by liquid scintillation counting, and values were normalized with cell numbers of each sample. The data are presented as the means ± SDs (n=10) and were analyzed by one-way ANOVA. (**D**) Western blot analysis of GLUT1 level in the indicated cells. Data are representative from two biological replicates. ****P < 0.0001, ns: not significant.

**Figure S4**


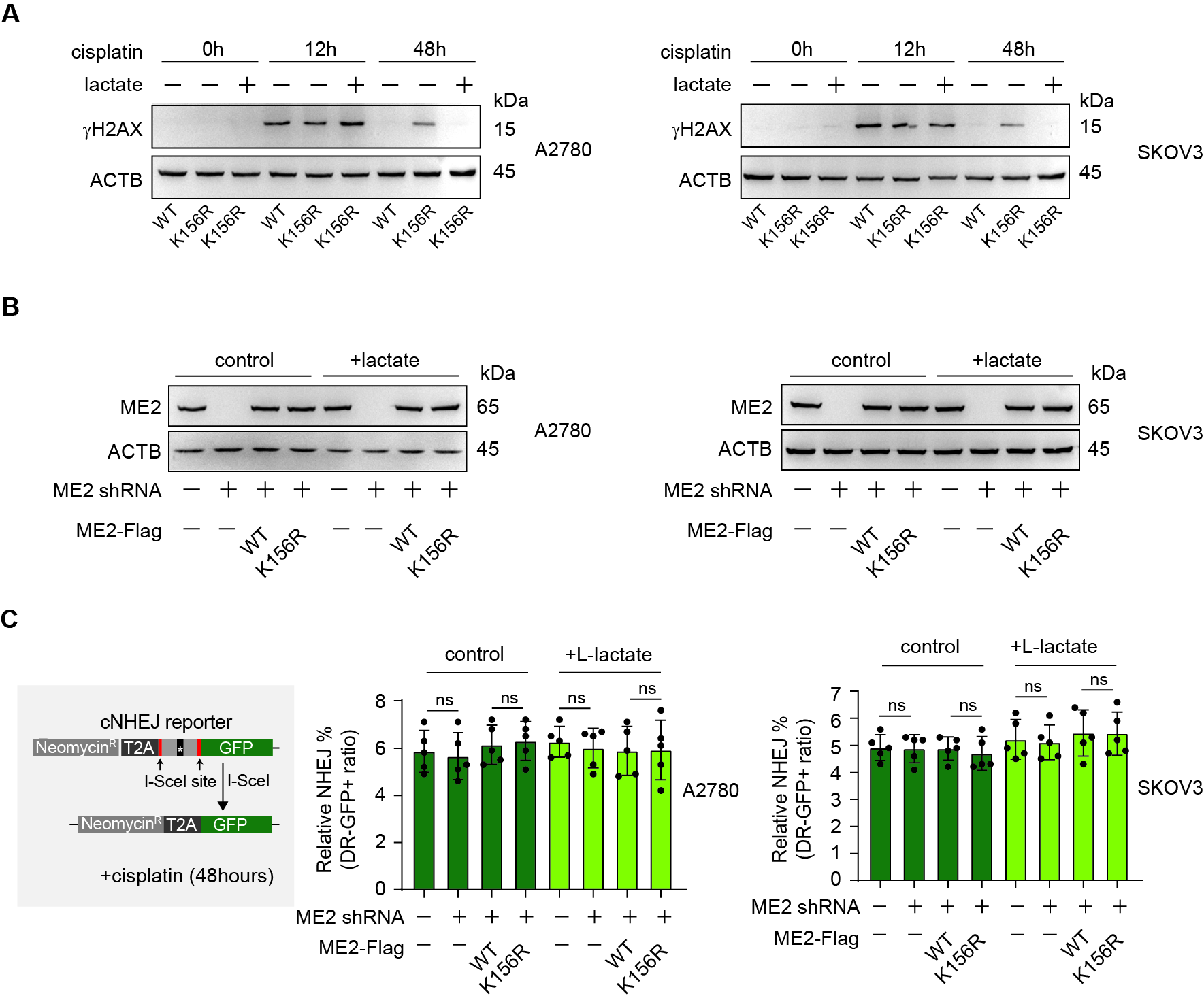


**Figure S4**. Related to Figure 4. Acetylated ME2 is important for homologous recombination repair via lactate. (**A**)Western blot analysis of total γH2AX level in A2780 (left) or SKOV3 (right) cells treated as indicated. L-lactate (20 mM) was added 24 hours after cisplatin treatment (1 µg/ml, 48h). Data are representative from two biological replicates. (**B**) Western blot analysis of indicated cell samples. (**C**) cNHEJ reporter assay was used to analyze NHEJ efficiency in cells by GFP expression using flow cytometry. L-lactate (20 mM) was added 24 hours after cisplatin treatment (1 µg/ml, 48h). Two-way ANOVA. ns: not significant.

**Figure S5**


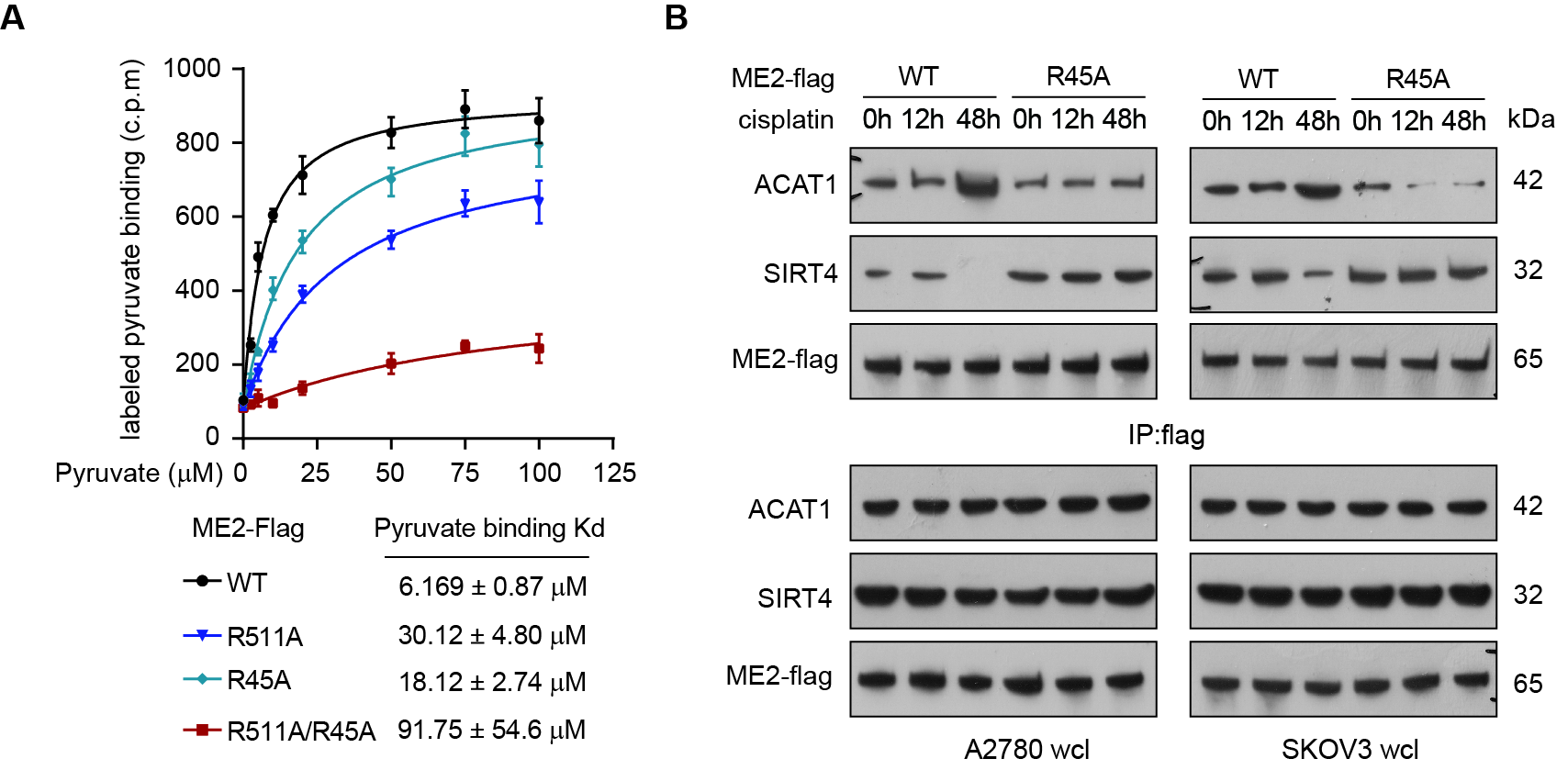


**Figure S5.** Related to Figure 6. Decreased pyruvate production resulting from low glucose levels promotes ACAT1 binding to ME2 and suppresses SIRT4 binding to ME2. (**A**) Kd values were determined via a 14C-labeled pyruvate binding assay. ME2 proteins purified from mammalian cells were incubated with increasing concentrations of 14C-labeled pyruvate. (**B**) Western blot of the indicated samples from ME2-flag WT- or mutant-expressing cells under cisplatin treatment, the blots shown are representative of three independent biological experiments.

**Supplementary Table S1**

The detailed results of the proteome-wide acetylation mass spec analysis

**Supplementary Table S2**

Clinical information of platinum-sensitive and platinum-resistant patients

**Supplementary Table S3**

Clinical information of patients for survival analysis

**Supplementary Table S4**

All key reagents used in this study

**Supplementary Table S5**

A detailed list of genes and the shRNA target sequence for acetyltransferase

**Supplementary Table S6**

A detailed list of genes and the shRNA target sequence for deacetylase
